# Antibody tests for diagnosing COVID-19: how relevant are they?

**DOI:** 10.11604/pamj.supp.2020.37.1.25822

**Published:** 2020-09-08

**Authors:** Muki Shehu Shey, Bey-Marrié Schmidt, Charles Shey Wiysonge

**Affiliations:** 1 Department of Medicine and CIDRI-Africa, University of Cape Town, Cape Town, South Africa,; 2 Cochrane South Africa, South African Medical Research Council, Cape Town, South Africa,; 3 School of Public Health and Family Medicine, University of Cape Town, Cape Town, South Africa,; 4 Department of Global Health, Stellenbosch University, Cape Town, South Africa

**Keywords:** Antibodies, COVID-19, serology testing

## Abstract

The current standards for detecting active coronavirus disease (COVID-19) infection are molecular tests by reverse transcription polymerase chain reaction, using swabs from the lower or upper respiratory tract. Because of the expertise required and the long turnaround time for the availability of test results, faster and easier point-of-care methods are necessary. The latter may include the detection of antibodies specific to COVID-19. We highlight a recent Cochrane review that assessed the accuracy of antibody tests for diagnosing COVID-19. The review shows that, at present, antibodies have little use in the diagnosis of COVID-19 within the first seven days from onset of symptoms. However, as time progresses, the sensitivity of the antibody tests increases. Antibody tests are more useful in detecting previous COVID-19 infection if used 15 days or more from onset of symptoms. Data presented in the review should be interpreted with caution as most studies (85%) recruited in-hospital patients and 11% recruited suspected COVID-19 patients, while only 4% recruited convalescent patients. This limits generalisability of the results to most settings.

## Commentary

Deeks and colleagues in an extensive Cochrane review presented an analysis on the diagnostic accuracy of antibody tests for the diagnosis of the coronavirus disease (COVID-19) [1]. COVID-19, caused by the severe acute respiratory syndrome coronavirus 2 (SARS-CoV-2), has become a pandemic after the first detection in Wuhan, China, in December 2019 [1]. Active SARS-CoV-2 infection is currently being detected by molecular tests by reverse transcription polymerase chain reaction (RT-PCR) using swabs from the lower or upper respiratory tract [2]. Because of the expertise required and the long turnaround time for the availability of results, faster and easier point-of-care methods are necessary, which may include the detection of antibodies specific to SARS-CoV-2 [1]. The main objectives of the review were to assess the diagnostic accuracy of different antibody tests from different studies and manufacturers for the diagnosis of active and or previous infection with SARS-CoV-2 in the community or healthcare facilities, and the accuracy of the antibody test for use in seroprevalence surveys.

A broad search strategy was used to identify potentially eligible studies. The searches were conducted in the Cochrane COVID-19 Study Register, COVID-19 Living Evidence Database from the University of Bern, and other electronic resources. Studies of all designs that provided estimates of accuracy for the different tests or from which accuracy of the tests could be computed were included. Studies for which data could not be extracted were excluded. Participants included in the studies were either symptomatic, asymptomatic, or those identified through contact tracing or community screening. Studies with less than 10 participants were excluded to limit bias and because such studies could give unreliable estimates for sensitivity and specificity. Antibody diagnostic tests evaluated included laboratory-based tests such as Enzyme-Linked Immunosorbent Assay (ELISA), chemiluminescence, and rapid diagnostic tests such as lateral flow assays. These tests were either those commercially available with regulatory approval, in-house assays or assays in development. The tests were either used to diagnose active or previous SARS-CoV-2 infection. A team of experienced review authors screened the search results to identify eligible studies, extracted data, assessed methodological quality of included studies in duplicate. In addition, they conducted assessment of heterogeneity and sensitivity analyses, and summarised key findings according to the strength of evidence for each test while highlighting the gaps in the evidence.

Different studies reported data on different antibody combinations for the diagnosis of SARS-CoV-2 infection including immunoglobulin G (IgG), IgM, IgA, IgG/IgM, IgG/IgA and total antibodies, with most studies evaluating IgG or IgM or combinations, and very few studies evaluating IgA and combinations. The IgG/IgM combination had a sensitivity of 30% for 1-7 days, 72% for 8-14 days, and 91% for 15 to 21 days. When studies were grouped together to analyse sensitivity, there was a huge variation by antibody type ranging from 20% to 100%. When stratified by time in days from onset of symptoms, sensitivity was generally low within the first 7 days and highest after 15 days. Irrespective of antibody type, false negative results were most frequent within the first 7 days and lowest after 22 days post onset of symptoms. True positive results were lowest within 7 days and highest after 22 days. Based on data presented in the review, antibodies have little use in the diagnosis of active SARS-CoV-2 infection and COVID-19 within the first 7 days from onset of symptoms. However, as time progresses, the sensitivity of the antibody tests increases. Antibody tests are more useful in detecting previous SARS-CoV-2 infection if used 15 days or more from onset of symptoms. Data presented in the review should be interpreted with caution as most studies (85%) recruited in-hospital patients, 11% recruiting suspected COVID-19 patients, while only about 4% recruited convalescent patients. This limits generalisability of the results to most settings.

According to the evidence presented in the Cochrane review by Deeks and colleagues, antibody detection is low within the first week of symptoms onset in COVID-19 patients [1]. Antibody detection gradually increases and is highest in the third week of infection up to 35 days. There is currently no reliable data on the sensitivity of these antibody tests after 35 days. In addition, these antibody tests do not seem to perform well in SARS-CoV-2 infected but asymptomatic patients or patients with milder symptoms. Based on current evidence, serology tests that detect antibodies to SARS-CoV-2 are not essential in the diagnosis and management of COVID-19 patients at the moment [3]. However, antibody tests are still essential in the management of children who have been found to have a hyperinflammatory syndrome related to SARS-CoV-2, where the polymerase chain reaction test is often negative, but positive for serology tests. In addition, serology testing for antibodies is still essential in the response to the pandemic in the research arena and in the general surveillance, to enable large-scale mapping of infections to show the rate of previous infection with the virus [3]. Other potential areas where the serology tests (especially the rapid point-of-care tests) may be useful include situations where access to molecular diagnostic tests is very limited and there is need for information on the extent of the outbreak, the geographical distribution, identifying hotspots, populations at risks and triaging symptomatic patients in community settings and testing of contacts of confirmed cases [4]. Evidence available up to date on the virology, immunology and transmission dynamics of the SARS-CoV-2 and COVID-19 have suggested that this is an extraordinary pandemic that requires extraordinary measures in response [4]. More research on the validity of antibody tests (and other rapid diagnostic tests) in different groups of participants/patients (general community, asymptomatic, symptomatic, and those presenting with symptoms but negative for molecular testing) and in patients beyond 35 days from symptoms onset is necessary to adequately define the utility of antibody tests and determine their sensitivity/specificity. Multicentre studies with proper definition of patient groups, study endpoints, serial testing within the same patients and adequate study designs with blinding of test results for the different tests compared are also necessary.
